# Changes in postural control in older adults: a five-year longitudinal study

**DOI:** 10.1038/s41598-026-36592-1

**Published:** 2026-02-06

**Authors:** Denisa Nohelova, Nicolas Vuillerme, Lucia Bizovska, Miroslav Janura, Zdenek Svoboda

**Affiliations:** 1 Department of Natural Sciences in Kinanthropology, Faculty of Physical Culture, Palacký University Olomouc, Olomouc, Czech Republic; 2 Université Grenoble Alpes, AGEIS, Grenoble, France; 3 Institut Universitaire de France, Paris, France

**Keywords:** Health care, Medical research, Physiology

## Abstract

At an advanced age, alterations in postural control are common however there is lack information related to high-functioning older adults. This study aimed to compare postural control in a bipedal stance in healthy, high-functioning older adults after 5 years.

Twenty-three older adults aged 65.7 ± 4.0 years were evaluated at baseline and after 5 years using the Activities-specific Balance Confidence Scale (ABC), the Tinetti balance assessment tool (TBAT) and posturography. Postural control in the bipedal stance was assessed for 30 s using two force platforms in firm surface with eyes open (EO) and closed (EC), and similarly in foam surface (FEO; FEC). During the 5-year follow-up, the ABC score significantly increased (*P* = 0.030), suggesting higher confidence in maintaining balance. The mean center-of-pressure velocity increased in both mediolateral (ML) and anteroposterior (AP) directions in the EO (*P* < 0.05) and FEO (*P* < 0.001). There were some significant changes in entropy measures for various procedures and directions. All these significant changes increased during five years period. Our results suggest that in high-functioning older adults the process of represents rather the adaptation, not an inevitable journey to “frailty” and the human body systems organize solutions with different levels of complexity.

## Introduction

The world population is aging as life expectancy increases due to healthcare improvements^1^. Therefore, the elderly population has been increasing rapidly in recent decades, linked to an increasing incidence of balance problems in older individuals. The ability to maintain postural stability during activities of daily living is essential for independent living. However, age-related declines in physiological functions may limit or prevent older adults from performing common daily activities and/or may even be the cause of accidents (e.g., falls) with subsequent health limitations^2^. Consequently, balance impairments tremendously impact the general health status and quality of life of older adults and increase the overall financial costs of medical care. Accordingly, the assessment of postural control has received considerable attention in recent decades; yet there are no universally applicable recommendations for clinical practice.

Postural stability, i.e., ability to control the position of the body in the space for the purpose of movement and balance, serves to maintain a static position, supports body coordination during dynamic position changes and enables to react to external and internal forces to avoid an unintentional fall^3^; and results from complex interactions between sensory systems (visual system, vestibular system, proprioceptive and exteroceptive somatosensory system), central nervous system (brainstem, spinal cord, cerebellum, sensory nuclei, (pre-)motor, and (pre-)frontal cortex) and motor system (motor units, muscles, tendons)^4^. Nevertheless, the structures and functions of the aforementioned systems are degraded by aging at an accelerated rate after the age of 60^5^, which causes alterations in the ability to maintain postural stability in older adults compared with young adults, as evidenced by several studies^6–8^. The ability to distinguish systems (vestibular, visual, proprioceptive, motor, etc.) disturbed in relation to imbalance using questionnaires, functional clinical tests, and testing batteries is limited^9,10^. Besides subjective clinical tests for balance evaluation, static posturography offers quantifiable analyses of postural control through center-of-pressure (COP) trajectory analysis^10^. Due to the measurement of whole-body dynamics, the stabilogram represents the sum of various neuromusculoskeletal components acting at different joint levels and underlying unique integrated systems^11^. Therefore, posturography has been proposed as a method to detect age-related changes in postural control at the sensory^12,13^, central^14–16^, and musculoskeletal levels^17,18^.

Moreover, in addition to the different ways of describing the COP trajectory based on the traditional linear approach, there are also approaches based on nonlinear procedures and dynamic system theory. While traditional methods of COP trajectory evaluation provide information about the gross amount of movement, they ignore the temporal structure of the COP trajectory and thus do not allow the quantification of possible temporal changes owing to the complex sensory-motor integration involved in postural control^19,20^. Nonlinear methods are, on the contrary, able to capture the temporal component of the variation in COP displacement in order to determine whether there is information about the underlying motor control processes and how motor behavior changes over time^11,21^.

In the assessment of postural control, entropy-based parameters of the COP are considered as one of the representatives of nonlinear procedures. However, because of the number of entropy derivatives, number of similar entropy algorithms, different input variable options for calculation, and contradictory behavior/results, the use of entropies can be quite confusing. Sequence entropies (i.e., approximate entropy (AppEn)^22^, sample entropy (SampEn)^23^ and multiscale entropy (MSE)^24^ quantify the regularity and complexity of the COP time series. Their values increase monotonously with the degree of randomness. Low entropy values correspond to regular or deterministic signals (i.e., they express lower complexity), and high entropy values correspond to an irregular or completely random signal (i.e., they express higher complexity)^25–27^. Whereas AppEn and SampEn estimate the randomness of a series of data over only a single timescale, the MSE algorithm was developed to account for the multiple timescales inherent in a time series^24–27^ to express the complexity of a biological signal. Furthermore, the index of complexity (CI), which is an estimation of the area under the MSE curve obtained by simply summing up the entropy values^28^, provides an overall quantitative measure to evaluate the loss of complexity hypothesis, as it allows the quantification of the integrated complexity of a biological signal across different timescales^29^. The loss of complexity hypothesis presumes that aging and disease cause the loss of complexity of structure, which translates into lower flexibility and higher rigidity of postural control^30^ and indicates that the number of dynamic interactions decreases^31^.

Although several authors have investigated the age-related influence on the dynamic structure of the COP trajectory during the quiet stance^31–36^, the age-related decline in postural control in older adults remains unclear. The major concern is that, traditionally, studies compare the motor performance of older adults with that of young adults or between different age subgroups (60–69 years old × 70–79 years old × 80–89 years old × 90^+^ years old), that is, different individuals with various characteristics (body configuration and composition). There is a lack of studies evaluating the performance of the same individuals longitudinally, i.e., after a certain period of time. Therefore, the age-related decline in postural control is not entirely clear, as is the behavior of the various variables describing the COP trajectory. From a dynamical systems perspective, aging-related changes in sensory processing, neuromuscular function, and central integration are known to alter not only the magnitude but also the temporal structure of postural sway. Previous studies have shown that aging is associated with alterations in the complexity of postural dynamics, reflecting sensorimotor reorganization and adaptive control processes^31,32^.

However, it should be noted that changes in nonlinear postural control metrics may reflect multiple underlying processes rather than a single mechanism. In addition to age-related alterations in sensorimotor integration, entropy and complexity measures can be influenced by task specificity and environmental constraints. Recent studies have shown that postural control adaptations depend strongly on the specificity of training and task conditions, with unstable surfaces eliciting distinct control strategies and patterns of variability^37,38^. Moreover, different neuromuscular control mechanisms have been identified for the regulation of static and dynamic balance^39^. Therefore, the interpretation of entropy- and complexity-based measures in aging populations requires careful consideration of task demands, environmental context, and potential adaptive processes.

To address the aging issue in static balance conditions, the purpose of the present study was to assess postural control in stance in older adults over a 5-year period and analyze their performance using both linear and nonlinear tools. A period of 5 years seems to be sufficient for age-related changes to appear, compared to a period of 3 years^31–36^, which seems likely to be too short to monitor the progression of aging.

We hypothesized that over the 5-years period, older adults exhibit changes in postural control characterized by increased postural sway and alteration in the temporal structure of center of pressure dynamics.

## Materials and methods

### Participants

In this longitudinal cohort study, participants were observed at baseline and 5 years later at follow-up. At baseline, the participants were recruited from local senior clubs and the University of the Third Age. The recruitment of participants for baseline started 1/3/2015 and ended 10/4/2015. All participants (*N* = 150) received the letter informing them of the possibility to participate in a follow-up study. The recruitment of participants for follow-up started 1/9/2020 and ended 1/10/2020. After telephonic interviews (to verify the inclusion and exclusion criteria), 50 older adults were invited for follow-up measurements. The main reasons for non-participation or exclusion were lack of response, unwillingness to participate, health deterioration that no longer met the inclusion criteria, time constraints, and limitations related to the COVID-19 pandemic. Of the 50 invited participants, 23 completed the follow-up assessment, resulting in a final research sample of 23 older adults (17 females, 6 males) aged 60–73 years. Consequently, the final sample represents a subgroup of relatively healthy, high-functioning older adults.

All participants were older than 60 years of age, had sufficient visual abilities (i.e., to read a written document), and could stand, walk, and perform activities of daily living without using auxiliary devices or assistance. They were free from neurological, vestibular, cognitive, or other diseases and medications affecting balance or gait and had not undergone lower limb or spine surgery in the previous 12 months. Their health status was checked during a detailed interview prior to the measurements.

The participants underwent the same experimental protocol at both time points. They wore comfortable sports shoes and clothing during all measurements. Only for postural stability measurement they were barefoot. All follow-up measurements were conducted using the same laboratory setup, instrumentation, and testing procedures as at baseline. The only procedural differences were enhanced hygienic measures implemented during the COVID-19 pandemic (e.g., disinfection protocols and use of personal protective equipment), which did not alter the testing conditions or task execution.

### Ethics approval

The study was approved by the Ethics Committee of the Faculty of Physical Culture, Palacký University Olomouc, Olomouc, Czech Republic, under reference numbers 24/2014 and 19/2020. All participants were informed of the aims of the study and the experimental protocol. All participants provided written informed consent prior to the measurements, in agreement with the Declaration of Helsinki.

Experimental protocol.

## Experimental protocol

### Clinical test and scale

After the participants arrived, their essential sociodemographic and anamnestic characteristics (sex, age, height, weight, body mass index (BMI), life situation, pain, illness, injury, medication, current health status, history of falls, etc.) were recorded. Subsequently, the Activities-Specific Balance Confidence Scale (ABC), that is, the scale of certainty of maintaining balance in specific activities^40^, which was translated into the Czech language in accordance with established international guidelines^41^, was completed. The ABC was developed as an extension of the Falls Efficacy Scale - International (FES-I) to provide a more detailed description of the difficulty and fear of falling when performing activities through more detailed item descriptors and a wider continuum of activity difficulty^40^. The ABC is a 16-item structured questionnaire scored from 0% (no confidence) to 100% (complete confidence), with a total score calculated by adding item scores and dividing by the total number of items. The total ABC score ranges from 0 to 100%, and the value of 67% has been proposed as reliable for predicting future falls^42^. The Tinetti balance assessment tool (TBAT) was incorporated to represent a standard clinical examination^43^. The TBAT uses a three-point scale (0, 1, and 2) and was evaluated in each section (maximum score in balance = 16 and gait = 12) separately and together (maximum score in total = 28). The higher the TBAT score, the lower the risk of falling. A TBAT score of less than 18 points indicates a high risk of falling^44^.

### Postural control during bipedal stance

The COP coordinates during the bipedal stance were recorded with two force plates AMTI OR6-5 (Advanced Mechanical Technology, Inc., Watertown, MA, USA; sampling frequency 200 Hz) embedded within the floor.

Postural control in the barefoot quiet stance (heels 15 cm apart, feet turned 10° outwards) with arms along the body was evaluated under four conditions in random order:

1. Standing on a firm surface with eyes open (EO).
2. Standing on a firm surface with eyes closed (EC).
3. Standing on a soft surface (foam pad Airex Balance Pad, AG, Sins, Switzerland 50 × 41 × 6 cm) with eyes open (FEO).
4. Standing on a soft surface with eyes closed (FEC).

During the eyes-open conditions, the participants were asked to stand as still as possible with their eyes open and look at the visual target (black square, 10 × 10 cm) located at their eye level on the wall at 1.5 m in front of them. Under soft surface conditions, a foam pad was placed on the force plates. Before we start the recording in all conditions, each participant remained in the testing positions for a few seconds to get familiar with it. Two trials were conducted for each experimental condition. Each trial lasted 30 s. The participants had a rest period of at least 60 s between trials. One participant refused testing in the FEC conditions.

### Data processing

The COP coordinates were computed using a custom MATLAB (MathWorks, Inc., Natick, MA, USA) algorithm based on the equations provided by the manufacturers of the force plates. Mediolateral (ML) and anteroposterior (AP) COP coordinates were considered for further analyses. Before computing the resulting variables describing the postural control, the 4th -order low-pass bidirectional Butterworth filter with a cut-off frequency of 10 Hz was applied to the COP coordinates. This filter setting is commonly used in posturography studies to attenuate high-frequency noise while preserving the physiologically relevant components of COP dynamics, including both velocity- and entropy-based measures^45,46^.

The following parameters describing the COP were calculated for each direction of the COP movement: the standard deviation (SD) of COP displacement and the mean COP movement velocity (VEL) were computed as a linear characteristic of postural sway^10^. The MSE was computed across 10 scales using standard parameter settings (embedding dimension m = 2; tolerance *r* = 0.15), which are commonly recommended for the analysis of physiological time series and postural control data^25^ using the software available on Physionet^24,28,47^. Using the same parameter values, AppEn and SampEn were also calculated^22,23^ to ensure methodological consistency across nonlinear metrics. In addition, CI was computed as area under MSE curve to provide an integrated measure of postural control complexity across multiple temporal scales.

For assessment of the effect of conditions also ratios EC/EO, FEO/EO and FEC/EO for all postural control variables were calculated.

### Statistical analysis

The statistical software Statistica 13 (TIBCO Software Inc., Palo Alto, CA, USA) was used for statistical data processing. The normal data distribution of all computed parameters was not confirmed by the Shapiro-Wilk test; therefore, nonparametric statistics were used further. The Wilcoxon matched-pairs test was used for all variables to compare the baseline and follow-up results. The level of statistical significance was set at α = 0.05. Effect size r for each case was computed in accordance with Rosenthal^48^.

## Results

### Somatic characteristics

Age and somatic characteristics during baseline and follow-up are described in Table 1. Although a small but statistically significant decrease in body height was observed at follow-up, body mass and BMI remained unchanged.

**Table 1:** Somatic characteristics at baseline and follow-up.

Variable	Baseline			Follow-up			*P*	*r*
	Median	Lower quartile	Upper quartile	Median	Lower quartile	Upper quartile		
Age (years)	64.4	63.1	69.1	69.9	68.5	74.5	< 0.001	0.62
Height (m)	1.66	1.61	1.71	1.65	1.60	1.71	0.003	0.47
Weight (kg)	74.4	62.8	81.7	74.9	63.3	81.2	0.927	0.01
BMI (kg.m^− 2^)	24.7	22.8	30.0	24.9	23.8	30.5	0.248	0.17

r, effect size; BMI, body mass index.

### Clinical scale and test

At the 5-year follow-up, ABC scores were significantly higher than at baseline, whereas TBAT scores did not differ significantly across time points (Table 2).

**Table 2:** Values of clinical tests at baseline and follow-up.

Variable	Baseline			Follow-up			*P*	*r*
	Median	Lower quartile	Upper quartile	Median	Lower quartile	Upper quartile		
ABC	91.9	85.0	96.9	95.6	89.4	98.8	0.030	0.32
TBAT gait	12.0	12.0	12.0	12.0	12.0	12.0	0.735	0.09
TBAT balance	16.0	16.0	16.0	16.0	16.0	16.0	0.465	0.26
TBAT total	28.0	28.0	28.0	28.0	28.0	28.0	0.953	0.01

r, effect size; ABC, The Activities-specific Balance Confidence Scale; TBAT, Tinetti balance assessment tool score.

### Postural control instance

In the EO condition, VEL significantly increased in both directions (*P* < 0.05 in both cases; *r* = 0.37 and *r* = 0.51 for ML and AP directions, respectively) during the follow-up measurement compared to the baseline (Table 3).

**Fig. 1:**
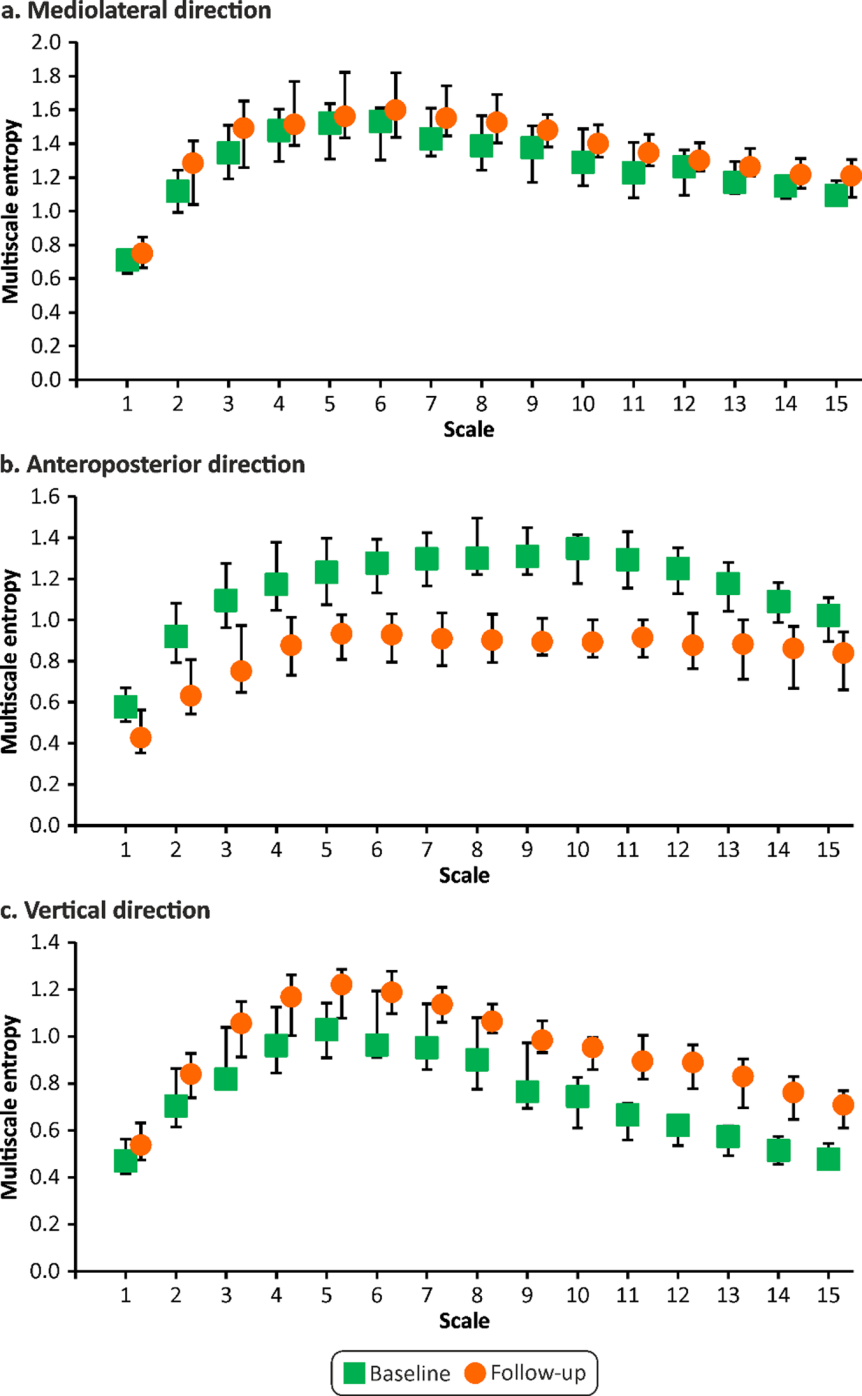
Multiscale entropy of center of pressure movement at baseline and follow-up.

MSE also significantly increased in the ML direction at all scales (*P* < 0.05; *r* ~ 0.31–0.41) and in the AP direction on a scale of 4–10 (*P* < 0.05; *r* ~ 0.32–0.38) (Fig. 1). The CI increased significantly in both directions: ML (*P* = 0.008; *r* = 0.39) and AP (*P* = 0.018; *r* = 0.35) during the follow-up measurement compared to baseline. Furthermore, in the ML direction, the AppEn significantly increased (*P* = 0.026; *r* = 0.33), as did the SampEn (*P* = 0.036; *r* = 0.31).

**Table 3:** Postural control in stance at baseline and follow-up.

Variable	Direction	Condition	Baseline			Follow-up			P	r
			Median	Lower quartile	Upper quartile	Median	Lower quartile	Upper quartile		
SD (mm)	ML	EO	1.8	1.5	2.1	1.8	1.3	2.3	0.394	0.13
		EC	1.8	1.4	2.2	1.8	1.3	2.1	0.484	0.10
		FEO	5.1	4.4	6.4	5.8	4.6	6.6	0.236	0.17
		FEC	7.6	6.2	8.4	6.4	5.4	7.4	**0.013**	0.37
	AP	EO	3.6	3.2	4.9	4.6	3.2	5.2	0.094	0.25
		EC	4.6	3.1	5.5	4.9	3.7	5.3	0.715	0.05
		FEO	6.3	5.4	7.8	8.7	7.4	10.2	**< 0.001**	0.61
		FEC	11.7	10.6	13.3	10.8	10.1	12.3	0.189	0.20
VEL (mm.s^− 1^)	ML	EO	3.4	2.8	4.2	3.7	2.9	4.8	**0.012**	0.37
		EC	4.4	3.5	5.6	3.8	3.4	5.4	**0.024**	0.33
		FEO	8.4	6.9	9.8	10.2	8.3	14.7	**< 0.001**	0.58
		FEC	15.1	12.0	20.4	12.5	10.8	17.0	0.115	0.24
	AP	EO	7.6	6.0	9.5	10.0	8.7	13.6	**0.001**	0.51
		EC	10.4	8.7	14.0	12.1	8.9	18.2	0.136	0.22
		FEO	16.3	12.6	17.8	25.7	19.3	31.5	**< 0.001**	0.62
		FEC	31.6	26.3	39.8	35.5	27.1	38.2	0.200	0.19
AppEn	ML	EO	0.109	0.093	0.157	0.132	0.099	0.196	**0.026**	0.33
		EC	0.130	0.108	0.166	0.122	0.112	0.178	0.394	0.13
		FEO	0.052	0.043	0.066	0.061	0.049	0.071	**0.006**	0.41
		FEC	0.070	0.053	0.076	0.063	0.058	0.074	0.758	0.05
	AP	EO	0.064	0.049	0.089	0.078	0.056	0.093	0.191	0.19
		EC	0.082	0.061	0.104	0.076	0.067	0.101	0.927	0.01
		FEO	0.071	0.057	0.088	0.077	0.064	0.099	**0.024**	0.33
		FEC	0.078	0.066	0.090	0.083	0.066	0.098	0.108	0.24
SampEn	ML	EO	0.147	0.109	0.174	0.179	0.130	0.208	**0.036**	0.31
		EC	0.177	0.149	0.216	0.183	0.125	0.247	0.670	0.06
		FEO	0.084	0.073	0.110	0.103	0.081	0.132	**0.001**	0.47
		FEC	0.109	0.094	0.152	0.112	0.100	0.127	0.808	0.04
	AP	EO	0.108	0.084	0.175	0.158	0.105	0.176	0.083	0.26
		EC	0.150	0.118	0.188	0.150	0.131	0.210	0.761	0.04
		FEO	0.134	0.120	0.186	0.155	0.126	0.207	**0.002**	0.46
		FEC	0.159	0.129	0.188	0.178	0.133	0.210	**0.016**	0.37
CI	ML	EO	2.6	2.0	3.4	3.3	2.7	3.7	**0.008**	0.39
		EC	3.3	3.0	3.9	3.6	2.7	4.5	0.627	0.07
		FEO	2.2	1.9	2.4	2.5	2.1	3.0	**< 0.001**	0.52
		FEC	2.7	2.3	3.3	2.7	2.5	3.0	0.592	0.08
	AP	EO	2.7	2.2	3.7	3.5	2.6	3.9	**0.018**	0.35
		EC	3.5	2.9	3.9	3.4	3.1	4.4	0.584	0.08
		FEO	3.2	3.0	3.7	3.5	3.1	4.1	**0.002**	0.46
		FEC	3.6	2.9	3.9	3.8	3.2	4.1	**0.017**	0.36

r, effect size; SD – standard deviation of centre of pressure displacement; VEL, mean centre of pressure velocity; AppEn, approximate entropy; SampEn, sample entropy; CI, index of complexity; ML, mediolateral direction; AP, anteroposterior direction; EO, firm surface with eyes open; EC, firm surface with eyes closed; FEC, soft surface with eyes closed; FEO, soft surface with eyes open.

In the EC condition, no statistically significant differences were found except for VEL in the ML direction (*P* = 0.024; *r* = 0.33), which decreased during the follow-up measurement compared with baseline.

In the FEO condition, SD in AP direction, the VEL in the ML and AP directions significantly increased (*P* < 0.001 in all cases; *r* = 0.61, *r* = 0.58, and *r* = 0.62, respectively) during the follow-up measurement compared with baseline. Significant increases were also observed in the MSE, CI, AppEn, and SampEn. MSE significantly increased in the ML and AP directions at all scales (*P* < 0.05; *r* ~ 0.39–0.53 and *P* < 0.05; *r* ~ 0.40–0.48, respectively) as CI in the ML and AP directions (*P* < 0.001; *r* = 0.52 and *P* = 0.002; *r* = 0.46, respectively), AppEn in the ML and AP directions (*P* = 0.006; *r* = 0.41 and *P* = 0.024; *r* = 0.33, respectively), and SampEn in the ML and AP directions (*P* = 0.001; *r* = 0.47 and *P* = 0.002; *r* = 0.46, respectively).

In the FEC condition, SD in ML direction significantly decreased (*P* = 0.013; *r* = 0.37) and in the AP direction, MSE in all scales, CI, and SampEn significantly increased (*P* < 0.05; *r* ~ 0.34-037 and *P* = 0.017; *r* = 0.36 and *P* = 0.016; *r* = 0.37, respectively) during the follow-up measurement compared to baseline.

The assessment of the effect of conditions for SD showed significant difference between baseline and follow-up only for AP direction in FEO/EO (baseline: 1.6, follow-up: 2.1, *p* = 0.006, *r* = 0.41) and in FEC/EO (baseline: 3.2, follow-up: 2.8, *p* = 0.0495, *r* = 0.30).

The significant differences for VEL in both ML and AP directions are summarized in Table 4. For EC/EO and FEC/EO there is decrease of values in the follow-up compared to baseline, while for FEO/EO there is significant increase.

**Table 4:** Postural control ratios of COP velocity in stance at baseline and follow-up.

Direction	Condition	Baseline			Follow-up			*P*	r
		Median	Lower quartile	Upper quartile	Median	Lower quartile	Upper quartile		
ML	EC/EO	1.3	1.1	1.5	1.1	1.0	1.2	**0.002**	0.47
	FEO/EO	2.3	2.0	2.6	2.7	2.2	3.7	**0.001**	0.49
	FEC/EO	4.4	3.7	5.3	3.7	2.9	4.5	**0.012**	0.38
AP	EC/EO	1.5	1.4	1.7	1.2	1.1	1.4	**0.006**	0.42
	FEO/EO	2.0	1.8	2.5	2.5	2.0	2.6	**0.026**	0.34
	FEC/EO	3.9	3.2	4.6	3.3	2.7	3.9	**0.011**	0.38

r, effect size; VEL, mean centre of pressure velocity; ML, mediolateral direction; AP, anteroposterior direction; EO, firm surface with eyes open; EC, firm surface with eyes closed; FEC, soft surface with eyes closed; FEO, soft surface with eyes open.Ratios derived from other variables did not show any significant difference between baseline and follow-up measurement except FEC/EO ratio derived from MSE scale 9 in ML direction (baseline 1.1, follow-up 0.9, *p* = 0.0495, *r* = 0.30).

## Discussion

The present study aimed to assess age-related changes in postural behavior in healthy, high-functioning older adults under various conditions over a 5-year period.

In general, results show that at older ages, individuals move away from their equilibrium point more quickly, requiring more frequent postural corrections to return to the equilibrium point, causing the COP movement to be more irregular, unpredictable, and variable; and this progression is pronounced mainly while standing on a soft surface with eyes open.

Although increased COP velocity is commonly interpreted as reduced postural efficiency, higher entropy and complexity reflect increased variability and flexibility in postural control strategies. These findings are not contradictory but rather represent complementary aspects of postural regulation. Increased sway velocity likely indicates more frequent corrective actions due to reduced sensorimotor precision, whereas elevated entropy suggests compensatory reorganization and greater adaptability of the postural control system. Thus, aging-related changes in postural control may involve a trade-off between mechanical efficiency and adaptive flexibility rather than a unidirectional improvement or decline^31^.

Among the anthropometric variables, a small but statistically significant decrease in body height (median difference: 1 cm) was observed at follow-up. Anthropometric characteristics, particularly body height, are known to influence postural sway by affecting the position of the center of mass and the length of body segments, which in turn may alter the magnitude of COP displacement and velocity. However, the observed reduction in height was minimal and likely reflects age-related factors such as spinal compression and postural changes rather than true alterations in body segment proportions.

Interestingly, ABC scores increased at follow-up, suggesting that participants maintained a high level of perceived balance confidence^49^. This finding suggests that perceived balance confidence does not necessarily parallel subtle age-related alteration in postural dynamics in high-functioning older adults. Several explanations may be considered. Previous studies have shown that balance confidence can remain stable or even increase with aging in physically active or well-functioning individuals, potentially reflecting successful behavioral adaptation, increased reliance on compensatory strategies, or recalibration of perceived abilities rather than objective balance capacity. In addition, psychological factors, such as habituation to balance challenges or changes in risk perception, may influence self-reported confidence over time. Although factors such as observation effects or response bias cannot be excluded, these interpretations remain speculative and should be regarded as hypotheses rather than definitive explanations, as they were not directly assessed in the present study.

The frequently used clinical test, TBAT, did not reveal any change in balance after five years. This finding suggests that older adults were able to fulfill the tasks, mainly from a quantitative point of view and gross motor skills (minor changes in movement quality are not tracked in TBAT) and it also denoted, that our participants had no major balance problems and were in good physical condition (as evidenced by their anamnesis). Furthermore, this testing battery is probably not sensitive enough to reveal age-related changes in healthy, active individuals and is more suitable for testing individuals with diseases or impairments.

Previous studies have demonstrated that body sway increases with age during quiet stance, particularly in the absence of visual input or on unstable surfaces^5,44,50–57^. The influence of the above-mentioned sensory conditions was also reflected in our findings, as evidenced especially by the gradual increase in VEL values in the EO, EC, FEO, and FEC conditions. This increase was consistent with the task difficulty. On the other hand, ratios between more demanding (EC, FEO and FEC) and EO conditions showed greater effect of proprioception (foam) and surprisingly lower effect of vision in follow-up compared to baseline. Similarly, we found age-related changes particularly when the eyes remained open. This may be surprising at first glance, as we might assume that more significant alterations would be observed under more demanding conditions (closed eyes). Nevertheless, owing to visual stimuli, the brain obtains information regarding changes in its surroundings and about changes related to the position of the body and its individual parts in space^58^. Therefore, visual information is of the utmost importance when determining and controlling the course of movement. Previous studies have reported that visual input can dominate other sensory systems involved in postural stability, and that humans primarily use and rely on visual stimuli during motor tasks^59^. Given that vision and other systems deteriorate in old age^60,61^, our finding is not unusual; on the contrary, it emphasizes the importance of visual stimuli and the impact of their deterioration on postural control in stance. Another interesting and practical finding is that age-related changes primarily affect postural control in older adults during activities of daily living in which they commonly use vision.

Furthermore, the eyes-open condition, in combination with the foam surface, revealed the most significant changes in postural control in stance. Therefore, a soft surface should be preferred in addition to the eyes-open condition for assessing age-related changes in postural control in stance. This recommendation aligns with the findings of Fujimoto et al.^62^, who also assessed that foam surface and the eyes-open condition highlighted age-related changes. Montesinos et al.^63^ further confirmed that this testing condition of “intermediate” difficulty discriminates better between more homogeneous groups (e.g., non-fallers versus fallers) than the least and the most challenging testing conditions.

Additionally, minor changes (in EC, only VEL decreased in the ML direction; in FEC, in the AP direction, CI and MSE in all scales increased) were observed in postural control during the eyes-closed condition, which could indicate that this testing condition may be too physically demanding to produce age-related differences in postural control and therefore might not be suitable for testing. Fujimoto et al.^62^ also did not find noticeable differences in FEC testing conditions among various age subgroups of older adults (55–64 years, 65–74, years and ≥ 75 years).

From a nonlinear perspective, a loss of complexity is expected with aging^29,31,64,65^. Since health requires the integration of control systems, feedback loops, and regulatory processes across multiple temporal and spatial scales that allow the organism to function and adapt to the demands of everyday life; aging (and disease) can be viewed as a breakdown of nonlinear feedback loops acting across multiple scales, leading to a loss of physiological complexity^45^. However, recent studies^66,67^ have failed to confirm the loss-of-complexity theory in healthy aging populations. Even though some studies reported reduction in complexity in the postural COP movement in older adults^11,19^, this manner was mainly observed among frail older individuals^33^ or in older individuals with visual and/or somatosensory impairment^31^, not in healthy aging, as there were no differences in CI and MSE detected between healthy young and healthy older adults or even the opposite trend – higher degree of complexity (maybe rather increased irregularity) was identified^67^. The reduction in complexity is more typical in frail older adults or individuals with diseases (e.g., multiple sclerosis and Parkinson’s disease). It appears to be caused rather by impairments in sensorimotor function^29,31,45,68,69^. In this case, the system is more unstable and less adaptable to perturbations^70^.

In our study, the entropy metrics generally increased in older adults during the 5-year follow-up. Overall, these findings suggest that COP movements are more irregular and complex at an advanced age, indicating an increased cognitive involvement in postural control and more frequent postural corrections^19,32^. Furthermore, in the AP direction in EO the changes were pronounced only in longer timescales, not in the short timescales, as is evident from the unchanged values of AppEn, SampEn, and MSE 1–3. This may indicate that automatic control remained untouched, whereas volitional control (longer timescales) was affected^32^. Therefore, MSE could provide a more detailed view of age-related changes in postural sway.

Taken together, our findings challenge the loss of complexity theory. COP movements became more complex and irregular over the 5-year period, particularly when participants stood upright with their eyes open on foam and firm surfaces.

This interpretation is consistent with previous studies indicating that reductions in physiological complexity are more strongly associated with frailty, sensory impairment, or pathological conditions rather than with healthy aging In relatively healthy older adults, preserved or even increased complexity may reflect flexible control strategies that compensate for subtle sensorimotor declines, as reported in both postural and gait studies^31–33^.

In relatively healthy older adults, preserved or even increased complexity may reflect flexible control strategies that compensate for subtle sensorimotor declines, as reported in both postural and gait studies^29,31,67^. Therefore, the loss-of-complexity hypothesis may be more applicable to vulnerable or clinical populations, whereas healthy aging appears to be characterized by context-dependent adaptations in postural control.

Although several parameters exhibited statistically significant changes over the 5-year follow-up, the magnitude of these changes was relatively small. Consequently, their clinical relevance to fall risk—particularly in high-functioning older adults—appears limited. For linear posturographic measures, increased COP velocity has been associated with a higher risk of falls in frail or clinical populations; however, clinically meaningful thresholds for quiet-standing COP metrics in healthy older adults remain poorly defined. Similarly, although changes in entropy- and complexity-based measures reflect alterations in postural control strategies, their direct relationship with functional performance or fall risk has not been firmly established. In the present cohort, clinical balance scores remained high and unchanged, suggesting that the observed posturographic changes likely represent subtle age-related adaptations rather than clinically overt balance impairments. Therefore, the detected differences should be interpreted as indicators of early or subclinical changes in postural control rather than direct markers of functional decline or increased fall risk.

Limitation and recommendations.

A major limitation of this study is the small sample size. Although 150 older adults were assessed at baseline, only 23 completed the follow-up assessment. This substantial attrition was primarily due to non-response, unwillingness to participate, failure to meet the inclusion criteria because of health deterioration (ascertained during telephone anamnesis), time constraints, and restrictions related to the COVID-19 pandemic. For these reasons, only 50 older adults were initially invited for the follow-up measurement. Additional COVID-19 restrictions imposed during the data collection period further limited testing capacity and required modifications of the study protocol, including exclusion of some planned measurements (e.g. physical activity monitoring and body composition assessment), and ultimately led to premature termination of the remaining measurements due to lockdown measures.

This high attrition rate introduces the potential for selection bias, as participants who remained in the study were likely healthier, more physically capable, and more motivated than those who withdrew or were excluded. Consequently, the observed changes in postural control may not reflect normative aging trajectories but rather aging-related adaptations in a relatively high-functioning subgroup of older adults, which limits the generalizability of the findings.

Follow-up data collection took place during the COVID-19 pandemic, which may have indirectly influenced participants’ physical activity levels, psychological well-being, and overall physical condition—factors known to affect balance performance and potentially contribute to inter-individual variability at follow-up. Nevertheless, all laboratory-based postural control assessments were conducted using the same equipment, setup, and testing procedures as at baseline, with the sole exception of enhanced hygienic measures. Therefore, while potential pandemic-related lifestyle effects cannot be entirely excluded, the comparability of the experimental procedures and measurement conditions between baseline and follow-up was preserved.

Another limitation is the lack of correction for multiple comparisons. Given the exploratory nature of the study and the relatively small sample size, no formal adjustment for multiple testing was applied, which increases the risk of type I error. Accordingly, the reported findings—particularly those with small effect sizes—should be interpreted with caution and considered hypothesis-generating rather than definitive.

In addition, the follow-up sample consisted predominantly of females (17 females vs. 6 males). Although sex imbalance is typical in older adult population in the Czech Republic^32^, sex related differences in neuromuscular and postural control may influence observed outcomes^32^.

Furthermore, the relatively low sensitivity of the TBAT for detecting subtle balance changes in healthy older adults represents another limitation. Future studies should consider more sensitive clinical assessments prospective designs linking posturographic changes to functional outcomes and fall risk.

## Conclusion

The present longitudinal study demonstrates that both linear and nonlinear characteristics of postural control in stance changed over a 5-year period in a group of healthy, high-functioning older adults. These changes were characterized by increased sway velocity and alterations in the temporal structure of COP dynamics. While the observed patterns may reflect adaptive or compensatory adjustments in postural control strategies, particularly in individuals who remain physically and functionally active, these interpretations should be made with caution given the small, highly selected sample and the exploratory nature of the analyses.

## Data Availability

The data presented in this study are available on request from the corresponding author.
